# Trends and protective factors of female genital mutilation in Burkina Faso: 1999 to 2010

**DOI:** 10.1186/s12939-015-0171-1

**Published:** 2015-05-08

**Authors:** Lana Clara Chikhungu, Nyovani Janet Madise

**Affiliations:** Division of Social Statistics and Demography, Centre for Global Health, Population, Poverty and Policy, Faculty of Social and Human Sciences, University of Southampton, Building 58, Social Sciences Academic Unit, University of Southampton, Highfield, Southampton, SO17 1BJ UK

**Keywords:** Female Genital Mutilation, Circumcision, Education, Burkina Faso

## Abstract

**Background:**

The practice of Female Genital Mutilation (FGM) is common in several African countries and some parts of Asia. This practice is not only a violation of human rights, but also puts women at risk of adverse health outcomes. This paper analysed the trends in the prevalence of FGM in Burkina Faso and investigated factors that are associated with this practice following the enactment of an FGM law in 1996.

**Methods:**

The study used the Burkina Faso Demographic and Health Survey (DHS) data sets from women aged 15 to 49 years undertaken in 1999, 2003 and 2010. Chi square tests were carried out to investigate whether there has been a change in the levels of FGM in Burkina Faso between 1999 and 2010 and multilevel logistic regression analysis were employed to identify factors that were significantly associated with undergoing FGM.

**Results:**

The levels of FGM in Burkina Faso declined significantly from 83.6% in 1999 to 76.1% in 2010. The percentage of women circumcised between the ages of 0 to 5 years increased from 34.2% in 1999 to 69% in 2010. Significantly more women in 2010 than in 1999 were of the opinion that FGM should stop (90.6% versus 75.1%, respectively). In 2010, the odds of getting circumcised were lowest amongst women that were born in the period 1990 to 1995 (immediately before the FGM law was enacted) compared to women born in the period 1960-1965 [OR 0.16 (0.13,0.20)]. There was significant variation of FGM across communities. Other factors that were significantly associated with being circumcised were education level, religion, ethnicity, urban residence and age at marriage.

**Conclusions:**

Although the prevalence of FGM has declined in Burkina Faso, the levels are still high. In order to tackle the practice of FGM in Burkina Faso, the government of Burkina Faso and its development partners need to encourage girls’ participation in education and target its sensitization campaigns against FGM towards Muslim women, women residing in rural areas and women of Mossi ethnic background.

**Electronic supplementary material:**

The online version of this article (doi:10.1186/s12939-015-0171-1) contains supplementary material, which is available to authorized users.

## Introduction

Female Genital Mutilation (FGM) also commonly referred to as female circumcision is the act of removing flesh from women’s genital area for cultural and non-therapeutic reasons [1]. FGM is commonly practiced in the North eastern and western Africa [2]. Women that have undergone FGM have increased health risks such as severe bleeding, development of urinary tract infections and cysts [3] and may have a higher likelihood of being affected by mental illnesses [4]. The practice of FGM does not only contribute to poor maternal and child health outcomes but is also a violation of human rights [5,6].

Between 100 and 140 million girls and women are estimated to have undergone FGM worldwide [5]. Amongst African countries where FGM is widely practiced, there is a variation in the prevalence of FGM. In Somalia, Guinea, Mali, Egypt, Sudan, Eritrea and Ethiopia the prevalence of FGM is more than 80%; in Senegal, Burkina Faso, Mauritania, Côte d’Ivoire, Chad, Central Africa Republic and Kenya, the prevalence rates range from 25% to 79%; and low prevalence rates of between 1% and 24% have been recorded in Ghana, Benin, Nigeria, Niger, and United Republic of Tanzania. In half of the countries where FGM is practiced, most of the girls are circumcised before they are five years old whilst in the rest of the countries cutting takes place between the ages of 5 and 14 [2].

In Burkina Faso, a country in West Africa, it is estimated that 76% of women have undergone circumcision [2]. Burkina Faso has some of the worst indicators of women and child health and development in the world. Approximately 10% of all children born die before reaching their fifth birthday, one-third of those who survive are stunted, and more than 300 women out of every 100,000 live births die as a result of pregnancy or childbirth complications [7]. Only about 29% of adults are literate and 48% girls are married before the age of 18 [8].

Although the prevalence of FGM remains high in Burkina Faso and many countries in Africa, significantly lower levels of FGM have been reported amongst women in younger age-groups compared to women in older age-groups suggesting a declining trend of the practice across generations [9-13].

There are four types of FGM. Type 1 involves the partial or complete removal of clitoris [14]. This type also has an Arabic name “Sunna”, which means duty [2]. In Somali where 97.9% of women have undergone FGM and the majority are Sunni Muslims, women support the continuation of the practice of the “Sunna” form of circumcision which is considered to be less harmful [11]. Across the countries that practise circumcision, Type 2 (excision) is also common and involves the removal of the clitoris and partial or complete removal of the labia minora [14]. Type 3 (infibulation) which is the partial or complete removal of any external genitalia with stitching or narrowing of the vaginal opening is the most severe form. Additionally, type 4 comprises all other harmful procedures to the female genitalia for non-medical purposes i.e. pricking, piercing, incising, scraping and cauterization [3].

Marriageability, religion and culture have been cited as some of the main reasons why the practice of FGM is persistent although no real benefits are known [3,11,15,16]. Indeed a study conducted in Burkina Faso across five districts reported that the majority of women (70%) could not cite a single benefit for undergoing FGM [17]. Such revelations support the role of peer convention and social networks in circumcision such that women may get circumcised just because others are also getting circumcised as suggested by Shell-Duncan *et al.* [18]. It may also be the reason why ethnicity emerges as an important factor associated with circumcision in many settings including Burkina Faso and Benin [2,17,19].

Despite marriageability being often reported as a possible reason for the continued practice of FGM [16], the link between marriage and undergoing circumcision is not well established and appears to vary across populations [11,20]. In Iraq, a study found that circumcised young women were more likely to be married compared to the uncircumcised [21]. The Social Convention theory first proposed by Mackie [22] postulates that in the context of resource inequality, FGM started as a means of securing a better marriage by signaling fidelity and later on became a prerequisite for marriage for all women. However, evidence shows that this is no longer the case in many settings [18,20]. Both FGM and early marriage are more common amongst populations with lower levels of education than the highly educated [8]. The association between FGM and early marriage has not been studied in Burkina Faso where approximately half of the girls are married before the age of 18.

The extent to which religion influences the practice of FGM is another matter of contention since the practice predates both Christianity and Islam [11,23,24]. A higher prevalence of FGM has been reported amongst Muslims compared to Christians and those of other religions in Burkina Faso and elsewhere [17,23,25,26]. The lower prevalence of FGM amongst Christians compared to Muslims in the studied populations could be because the Christian missionaries opposed the practice [23].

Previous studies have also shown a lower prevalence of FGM and greater support for the discontinuation of FGM amongst the highly educated women compared to those of lower levels of education in Burkina Faso and other countries [9,15,19-21]. Women’s education improves their position in society through increased control of resources which is influential in the wellbeing of children including survival and educated women are less likely to have their daughters circumcised [27,28].

Considerable work has been undertaken at international, national and community levels over many years to tackle FGM with some minimal impact [2,11,29]. A number of international and regional treaties and consensus communiqué have been documented such as the Convention on the Elimination of all Forms of Discrimination against Women (CEDAW), Convention of the Rights of the Child, and the African Charter on the Rights and Welfare [30]. A majority of countries have ratified a number of these treaties and have enacted criminal laws that provide penalties for participating in FGM practices and many provide protections and remedies to those that have undergone the procedure [31]. It has also been established that women that have participated in anti FGM interventions or have been exposed to anti FGM media are more likely to favour discontinuation of the practice signifying the importance of information and public awareness in changing women’s attitudes towards FGM [15,32].

Burkina Faso is amongst the few countries that enacted an FGM law in 1996. Burkina Faso also established a comprehensive process which encouraged the adoption of the legislation and facilitated its enforcement. This included the establishment of a programme on the campaign to end excision in 2002, capacity development of various stakeholders such as judges, lawyers and the police and education and awareness campaigns about the law and harmful effects of FGM [31]. It is crucial however that the trend in FGM in Burkina Faso be quantified to evaluate the impact of the FGM laws and policies, but also if a declining trend is established, to identify potential factors that may explain the declining trend. Such information would be vital for other countries that are in the process of enacting FGM policies and laws.

The main objectives of this paper were (i) to estimate the trends in the prevalence of FGM in Burkina Faso between 1999 and 2010 (ii) investigate the factors that are associated with FGM in Burkina Faso and (iii) if evidence suggests a decline of FGM between 1999 and 2010, identify factors that may have contributed to the decline.

## Methods

The analysis used data from women aged 15 to 49 years from the Burkina Faso Demographic and Health Surveys (DHS) of 1999, 2003 and 2010. The sample sizes for each of the survey years are shown in Table 1. The table also shows the distribution of women by selected characteristics likely to be associated with undergoing FGM as discussed in the introduction. The analysis of FGM was restricted to women excluding daughters for ease of comparison over the three time points because estimates indicated that nearly all women that had undergone FGM had done so before the age of 15. Since Burkina Faso implemented its FGM law in 1996, when examining prevalence of FGM among 15–49 year olds, rates for women born before 1990 can be seen as a pre-FGM law values, whereas rates for women born after 1990 are at the start of the impact of the FGM law.

**Table 1:** **Socio-demographic characteristics of Burkinabe women aged 15–49, 1999, 2003 and 2010 DHS data sets**

**Sample characteristics**	**1999**	**2003**	**2010**
Number of women interviewed	6,445	12,477	17,807
% of women that are Muslim	58.1	61.2	62.3
% of women that are Christian	28.4	28.8	30.3
% of women that are of traditional or other religions	13.6	10.1	7.7
% of women residing in rural areas	83.2	78.4	72.9
*% of women in each of the Ethnic groups* ^*1*^			
Mossi	58.8	56.6	52.5
Bobo, Dioula, Senoufu	7.5	12.0	10.0
Fulfuldé, Peul, Touareg, Bella	6.9	7.4	10.3
Gourmatché	8.2	6.9	6.9
Gourounsi and Bissa	6.9	3.4	8.5
Lobbi and Dagara	4.8	9.9	4.9
Others	7.1	3.8	7.0
Mean age of women	28.4	28.8	28.8
Mean age at marriage	17.3	17.4	17.6
% of women with primary or higher education*	14.2	19.7	26.0
% of women circumcised^2^*	83.6	79.2	76.1
% of women who would like circumcision to stop^3^*	75.7	81.3	90.6
*% of women who would like circumcision to stop by Birth cohort*			
1950-1959	74.7	81.4	
1960-1969	77.8	82.6	90.0
1970-1979	76.1	82.3	91.6
1980-1989	73.1	79.6	91.1
1990-1999			89.4
% of women who would like circumcision to stop* by education level			
No education	73.4	79.4	89.4
Primary education or higher	86.9	88.3	94.3
% of women who would like circumcision to stop* by religion			
Muslim	72.9	76.6	88.4
Christian	84.4	90.3	95.6
Traditional or other religions	68.5	80.3	90.3

Sources of data: DHS 1999, 2003, 2010 *the percentage differences across the three years were statistically different, Chi Square p value < 0.001.

^1^The variable on ethnicity was derived by combining smaller ethnic groups based on close proximity. An ethnic map of Burkina Faso which was used for this purpose is provided in Additional file 1.

^2^The sample used to calculate estimates of women circumcised does not include women with missing cases

^3^5.2% of women that said don’t know or it depends were set to missing so as to only compare the change in the percentage who would like circumcision to stop and those who would like circumcision to continue.

The dependent variable “being circumcised” is binary and was coded 1 if the woman was circumcised and 0 otherwise. No distinction was made by the type of circumcision since the interest of the study was to analyse the prevalence of any type of FGM and only small a percentage of women reported to have had their vaginal area sewn; 2.8% in 1999, 2.0% in 2003 and 1.2% in 2010. Bivariate analysis was undertaken to identify socio-economic and demographic factors that were significantly associated with being circumcised based on factors discussed in the introduction.

We explored the need for a multilevel model by estimating community^a^ (group of households) level variances in the 1999, 2003 and 2010 surveys and they were significant; 2.01 (standard error 0.285), 1.76 (standard error 0.165) and 1.76 (standard error 0.139) respectively.

Multilevel modelling ensures that estimates are robust in cases where data are hierarchical and the factor being studied varies significantly at a higher level. The final analysis used multilevel logistic regression modelling to estimate the odds ratios (OR) of a woman getting circumcised or not, after taking into account the socioeconomic and demographic factors that influence the likelihood of getting circumcised that are presented in Table 1. The fixed effects estimates are representative of the community level. A p-value of less than 0.05 was used to decide which variables were significant in both the bivariate and multivariate analysis.

**Table 2:** **Percentage of women in Burkina Faso experiencing FGM by age-group and year of interview**

**Age at circumcision**	**1999**	**2003**	**2010**
0 to 5 years	34.2	58.6	69.0
6 to 10 years	30.5	26.4	24.9
11 to 15 years	5.5	5.5	5.2
16 years and more	1.0	0.6	0.6
Don’t know	28.9	8.9	0.3
	N = 4616	N = 9524	N = 12923

Sources of data: DHS 1999, 2003, 2010, Chi square test P value <0.001.

### Modelling framework

We used the logit link ${ \log}_e\left(\frac{\pi_{ij}}{1-{\pi}_{ij}}\right)$, a function that models the probability that a woman *i* in community *j* is circumcised. We fitted a two level random intercept model, with the woman as the first level and the community as the second level. The two-level random intercept model for woman *i* nested within a community *j* may be represented as follows;

$$ { \log}_e\left(\frac{\pi ij}{1-\pi ij}\right)={\beta}_o+{\beta}_1{x_1}_{ij}++{\beta}_2{x_2}_{ij}+\kern1.5em \dots +{\beta}_6{x_6}_{ij}+{u}_{oj} $$

$$ {u}_{oj}\sim N\left(0,{\sigma}_{\mu {0}^2}\right) $$

Where *x*_1_ to *x*_6_ represent socio-economic explanatory variables for the probability that woman *i* in community *j* is circumcised. *β*_0_ is the overall intercept and *β*_1_ to *β*_6_ are coefficients for the explanatory variables *x*_1_ to *x*_6_. *U*_0*j*_ is the community-level random effect, which represents the variation of the likelihood of being circumcised for women from different communities and is assumed to be normally distributed with mean equal to 0 and variance equal to σ_μ0_^2^.

## Results

### Descriptive analysis results

The prevalence of FGM in Burkina Faso was very high, estimated at 76.1% in 2010 (Table 1). This however was significantly lower than the rate of FGM from the 1999 DHS data (83.6%) indicating a 7 percentage points reduction between 1999 and 2010. There were similarities across the three time points in the women’s mean age and the mean age at marriage. The percentage of women with primary education or higher significantly increased from 14.2% in 1999 to 26% in 2010 (p value <0.001). Table 1 also shows that the percentage of women who would like female circumcision to stop was very high and this increased from 75.7% in 1999 to 90.6% in 2010. The percentage of women who would like circumcision to stop also increased between the two time points in all birth cohorts where a comparison could be made, across the education levels and across religion groups. The percentage of women who would like circumcision to stop was significantly higher amongst women educated to the primary level or above than amongst women with no education and was significantly higher amongst Christian women than amongst Muslim women in all the three survey years, p value <0.001. The percentage differences of women who would like circumcision to stop across birth cohorts in each of the survey year were statistically significant but small.

The majority of FGM was performed by the traditional circumciser and this trend was increasing; 86.3% in 1999, 88% in 2003 and 97.2% in 2010 (p value <0.001). Only a small percentage of women reported to have had their vaginal area sewn and the percentage had declined over the study period 2.8% in 1999, 2.0% in 2003 and 1.2% in 2010 (p value <0.001).

An analysis of women’s perceptions on why female circumcision is practiced found that in 2003, 19.3% of women reported that circumcision is required by religion whilst in 2010 the percentage of women stating the same reason was 17%. The estimate for 1999 could not be obtained due to differences in the way the data was coded.

Table 2 shows the distribution of circumcised women in Burkina Faso by the age when circumcision was undertaken. The majority of women were circumcised by the age of 15 years. Worryingly, the data indicated that between 1999 and 2010, the percentage of women circumcised between the ages of 0 to 5 years increased from 34.2% in 1999 to 69% in 2010.

**Table 3:** **Percentage of women circumcised in Burkina Faso by socio-economic and demographic characteristics**

**Socio-economic characteristics**	**1999 (N = 5522)**	**N**	**Chi square P value**	**2003 (N = 12059)**	**N**	**Chi square P value**	**2010 (N = 17008)**	**N**	**Chi square P value**
*Birth cohort*									
1950-1959	87.5	1,078	<0.001	86.3	1,252	<0.001			
1960-1969	85.4	1,665		84.2	2,856		89.3	2,493	<0.001
1970-1979	83.1	2,228		81.0	3,702		85.1	4,254	
1980-1989	79.0	1,474		72.8	4,667		74.8	6,210	
1990-1995							60.7	4,130	
*Education Level*									
No education	84.2	5,240	0.005	81.3	9,901	<0.001	80.7	12,469	<0.001
Primary or Higher	80.3	1,205		70.8	2,573		63.7	4,611	
*Residence*									
Urban	84.6	1,651	0.37	75.9	3,014	<0.001	69.0	5,368	<0.001
Rural	83.4	4,794		80.2	9,463		78.7	11,719	
*Age at Marriage*									
Early (Before age 18)	85.8	3,416	0.03	82.9	6,219	0.04	81.0	7,913	0.09
After age 18	83.2	1,818		81.0	3,823		79.7	6,054	
*Religion*									
Muslim	88.4	3,601	<0.001	84.6	6,982	<0.001	81.7	10,239	<0.001
Christian	74.5	1,871		69.5	3,723		65.0	5,251	
Traditional and other religions	84.1	740		76.5	1,516		75.9	1,423	
*Ethnicity* ^*1*^									
Mossi	84.3	3,847	<0.001	81.2	6,552	<0.001	78.8	8,948	<0.001
Bobo, Dioula, Senoufu	92.5	334		87.2	1,149		69.4	802	
Fulfuldé, Peul, Touareg, Bella	92.0	416		75.7	862		73.1	1,607	
Gourmatché	69.6	460		70.6	698		64.5	1,048	
Gourounsi and Bissa	66.3	435		51.0	575		71.1	1,466	
Lobbi and Dagara	87.6	338		79.0	1,591		76.4	1,178	
Others	89.0	614		79.2	1,027		80.1	2,008	

Sources of data: DHS 1999, 2003, 2010 ^1^The Mossi make up 50% of all ethnicities.

Previous research on FGM reveals that the prevalence of FGM varies by socio-economic factors such as education level, urban/rural residence, religion and age. An investigation is therefore made of the variation of FGM across these socio-economic and demographic factors. The birth cohort is used instead of age, so as to investigate the impact of the enactment of the FGM which took place in 1996. Results presented in Table 3 indicated that the percentage of women circumcised was significantly higher amongst women with no education than women with primary education or higher, those from rural areas compared to those from urban areas, Muslim women compared to Christians and those of other religions and amongst those of Bobo, Dioula, Senoufu, Fulfuldé, Peul, Toaureg, Bella ethnicity compared to the Gourounsi and Bissa (see Additional file 1 for an ethnic map of Burkina Faso). The association between FGM and urban/rural residence was statistically significant in 2003 and 2010 but not in 1999.

**Figure 1:**
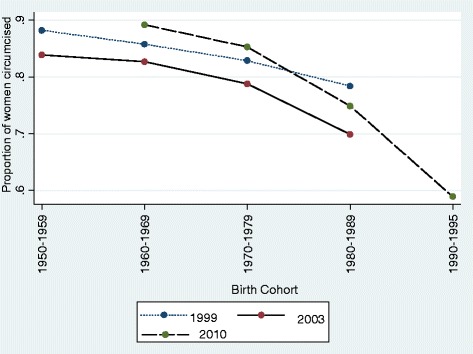
Proportion of Burkinabe women circumcised by birth cohort and year of interview.

In all the three survey years, the percentage of women circumcised was significantly lower amongst women in the recent birth cohorts than in the older cohorts. In 1999 and 2003, the lowest percentage of women circumcised was amongst women born in the period 1980 – 1989 and was highest amongst women born in the period 1950 to 1959 and in 2010 the lowest percentage of women circumcised was amongst women born in the period 1990–1995 and highest amongst women born in the period 1960–1969. A comparison of circumcision rates for women of similar birth cohorts in the three surveys indicated that the amongst women of 1970–1979 birth cohort, the percentage of women that reported to be circumcised was significantly lower in 2003, 78.8% (95% CI 77%.4, 80.1%) than in 1999 82.9% (95% CI 81.2%, 84.5%) and amongst women of 1980 – 1989 birth cohort, the percentage of women that reported to have been circumcised was significantly lower in 2003 69.9 (95% CI 68.5, 71.2) compared to 1999 78.4% (95% CI 76.1, 80.7). This is further illustrated in Figure 1.

**Figure 2:**
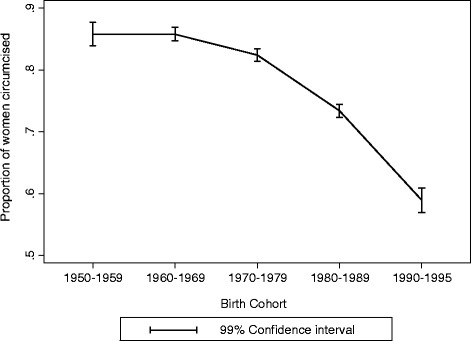
Proportion of Burkinabe women circumcised by birth cohort, data from 1999, 2003 and 2010 Burkina Faso demographic health surveys.

These findings suggest that some women who reported having undergone circumcision in 1999 may have changed their status to not circumcised in the 2003 survey as a result of the implementation of the law which was enacted in 1996. Evidence of women changing their FGM status following an enactment of an anti FGM law has been reported in a longitudinal study conducted in Ghana [33]. When data from the three surveys were combined, the percentage of women that reported to be circumcised declined significantly in each of the successive cohorts after the 1960–1969, (Figure 2).

### Regression results for the odds of being circumcised in Burkina Faso

The results of the fixed effects of the multilevel logistic regression for the odds of being circumcised are given in Table 4 and the results of the random effects are presented in Table 5. The fixed effects results are community specific. The odds of undergoing FGM were higher amongst women in the older cohorts (1950–1959, 1960–1969) compared to women from the more recent cohorts (1980–1989 and 1990 to 1995). In 2010 the odds of getting circumcised were lowest amongst women that were born in the period 1990 to 1995 compared to women born in the period 1960–1969 [OR 0.16 (0.13, 0.20)]. There was a significant association between ethnicity and being circumcised but the extent of the relationship varied over the three surveys for some of the ethnicities. Women of Gourounsi and Bissa ethnic background had consistently lower odds of being circumcised compared to women of Mossi ethnicity in 1999, 2003 and 2010; odds ratios [0.43 (0.26, 0.70)], [0.33 (0.22, 0.50)], [0.66 (0.51, 0.85)] respectively, whilst women of Fulfuldé, Peul, Touareg, Bella and Gourmatché ethnicities had lower odds of being circumcised compared to women of Mossi ethnicity in 2003 and 2010 but not in 1999. The likelihood of being circumcised was lower for women of Lobbi and Dagara ethnic background than women of Mossi ethnicity in 2003 but not in 1999 and 2010. The odds of getting circumcised were higher amongst Muslim women and those of traditional or other religions compared to Christian women in all the three surveys. On average the odds of being circumcised for Muslim women were double those of Christian women in all three surveys and the magnitude of the odds of being circumcised for Muslim women compared to Christian women showed an increasing trend between 1999 and 2010; in 1999 1.79 (1.35, 2.36), in 2003 1.86(1.56,2.33) and 2010 2.13 (1.86, 2.45). The association between women’s education level and being circumcised and that of age at marriage and being circumcised was only significant in the 2010 survey. Women with primary or higher levels of education were less likely to be circumcised compared to women with no education [OR 0.0.80 (0.69, 0.92)] and women that married after the age 18 were less likely to be have undergone FGM compared to women that married before age 18 [OR 0.85, (0.76, 0.94)].

**Table 4:** **Odds ratios of experiencing FGM and 95% Confidence Intervals (CI) amongst women aged 15–49 years in Burkina Faso, 1999, 2003 and 2010**

**Variable**	**Odds of being circumcised, 1999**	**95% CI**	**Odds of being circumcised, 2003**	**95% CI**	**Odds of being circumcised, 2010**	**95% CI**
*Birth Cohort *(Reference is 1950–1959 in 1999 and 2003 and 1960–1969 in 2010)						
1960-1969	0.76	(0.56,1.03)	0.80	(0.64,1.00)		
1979-1979	0.67	(0.50,0.89)*	0.58	(0.47,0.72)**	0.64	(0.54,0.77)**
1980-1989	0.53	(0.35,0.79)*	0.42	(0.34,0.53)**	0.31	(0.26,0.36)**
1990-1995					0.16	(0.13,0.20)**
*Age at Marriage (Reference is Before age 18)*						
18 Years or more	0.84	(0.68,1.05)	0.90	(0.79,1.03)	0.85	(0.76,0.94)*
*Ethnicity*: Reference is Mossi						
Bobo, Dioula, Senoufu	0.88	(0.40,1.95)	1.19	(0.84,1.71)	0.61	(0.45,0.82)*
Fulfuldé,Peul,Touareg,Bella	0.99	(0.54,1.80)	0.62	(0.45,0.86)*	0.38	(0.30,0.48)**
Gourmatché	1.49	(0.72,3.06)	0.52	(0.33,0.79)*	0.32	(0.23,0.45)*
Gourounsi and Bissa	0.43	(0.26,0.70)*	0.33	(0.22,0.50)**	0.66	(0.51,0.85)*
Lobbi and Dagara	1.96	(0.95,4.04)	0.65	(0.46,0.92)*	0.99	(0.70,1.40)
Other ethnicities	0.88	(0.55,1.41)	0.84	(0.59,1.20)	0.81	(0.64,1.03)
*Education level*: Reference is no education						
Primary education or higher	0.99	(0.71,1.38)	0.97	(0.79,1.18)	0.80	(0.69,0.92)*
*Residence* : Reference is Urban						
Rural	0.67	(0.39,1.17)	1.03	(0.72,1.49)	1.61	(1.20,2.15)*
*Religion*: Reference is Christian						
Muslims	1.79	(1.35,2.36)**	1.86	(1.56,2.33)**	2.13	(1.86,2.45)**
Traditional and other religions	1.81	(1.23,2.66)*	1.34	(1.06,1.69)*	1.44	(1.14,1.82)*

Sources of data: DHS 1999, 2003, 2010 *P value <0.05, **P value <0.001.

**Table 5:** **Results of the random parts of the models on FGM at community level, 1999, 2003 and 2010**

**Model**	**Variance**	**Standard error**	**Wald’s test statistic chi square P value**
1999	2.17	0.36	<0.001
2003	1.71	0.18	<0.001
2010	2.06	0.18	<0.001

Sources of data: DHS 1999, 2003, 2010.

The random effects shown in Table 5 indicate a significant variation in the odds of women getting circumcised across communities in all three surveys even after the inclusion of socio-economic variables.

## Discussion of findings

Although the prevalence of FGM among women aged 15 to 49 years is declining in Burkina Faso, the rates still remain very high. The increase in the percentage of women that are circumcised by the age of five is worrying and seems to suggest that as a result of the introduction of programmes and the law prohibiting FGM in 1996 people may have resorted to circumcising their daughters at younger ages when it may be difficult to get caught. The same reason may explain why the percentage of girls that are circumcised by a traditional circumciser has increased between 1999 and 2010, since the law may not support the medicalization of FGM. In Senegal the outlawing of FGM in 1999 resulted in multiple responses from individuals of the same extended families which included driving the practice underground [18]. This suggests that to fully tackle FGM, FGM legislation needs to be supported by programmes that carefully monitor the potential for underground activities [18].

Overall Burkinabe women in the younger birth cohort (1990–1995) had the lowest odds of being circumcised. The youngest group of women in this birth cohort were only a year old in 1996 when the FGM law was enacted in Burkina Faso whilst the oldest group should have been aged 6. It is possible that the enactment of the FGM law may have prevented some of the girls from undergoing FGM. The FGM law may also have had an impact amongst women of the 1980 to 1989 birth cohort amongst which sharp declines were registered compared to the 1970–1979 birth cohort. These findings are in line with those from previous studies that report low levels of circumcision amongst younger age groups [9,32].

With regards to a shift in opinion towards FGM, although overall there is a significant increase in the percentage of women that would like circumcision to stop, the analysis within each survey year showed only small percentage differences suggesting that women of different generations might influence each other’s opinion on FGM. This supports the role of peer convention theory, social networks and mother influence on a child’s FGM view or status [18,27].

The percentage of women that have primary education or higher increased between 1999 and 2010 and the odds of being circumcised amongst women with primary education or higher were lower than in women without education in 2010. The importance of education in lowering the likelihood of FGM in Burkina Faso has been reported in previous studies [17,19,26]. Considering that in most cases, circumcision takes place before girls start school, the relationship between education and FGM is expected to be indirect and its impact to be realized in the long-term. For example educated women may not subject their daughters to FGM either as a result of socialization with women who may have strong opposition to the practice of FGM or as a result of better exposure to interventions against FGM [2,23].

In Burkina Faso, Muslim women are more likely to be circumcised and to perceive that FGM should continue compared to Christians and women of traditional or other religions. This finding is similar to findings from previous studies conducted in Burkina Faso and elsewhere [11,17,25-27]. Although Islam does not recommend or promote FGM, it is suggested that Muslims may have culturally interpreted the practice as part of their religious identity over time [26].

The likelihood of getting circumcised was significantly higher amongst women of Mossi ethnicity compared to women of other ethnicities across the three survey years. A lower likelihood of female circumcision amongst women of Gourmatche ethnicity has been reported in a previous study whilst in another study significantly higher levels of circumcision in other ethnicities than those found in this study have been reported which may be attributed to differences in how the ethnicity variable was categorized [17,19].

In 2010, women who married early (before age 18) were more likely to be circumcised than women who married at the age of 18 or later, in line with findings from Iraq [21]. Recent evidence does not support the social convention theory that circumcision increases the chance of getting married but early marriage and FGM appear to be independently influenced by factors such as residence, education, ethnicity and religion and these factors may influence males’ preference for circumcised women [11,20,34].

Also in 2010 women from urban areas were less likely to be circumcised than women from rural areas whilst in 1999 and 2003 there were no significant differences between the two population groups. Over time one may expect improvements such as better access to information and education and such improvement may have favoured urban areas better than rural areas. No significant differences in the probability of getting circumcised were identified between women from urban and rural areas in a previous study conducted in Burkina Faso using the 2003 DHS data [19].

One limitation to this study is that the reliability of information of women’s circumcision status may be questionable since it was based on women’s reports rather than clinical data. The validity of the data might be challenged even more since they were collected after the FGM law was enacted. Evidence suggests women may change their report of FGM status after FGM laws are enacted [33]. Other studies reveal inconsistencies between self- reported and clinically determined FGM status [10,35] although the study by Elmusharaf only found inconsistency in the type of circumcision reported but not the FGM status [35]. Our findings suggest that women in the studied samples may have changed their report on their FGM status between survey years. However, the analysis of the levels of FGM by birth cohort from a sample of women from all surveys showed a significant decline in FGM across birth cohorts despite the possible underreporting as illustrated in Figure 2. The decline in the levels of FGM in Burkina Faso may be attributed to increases in the education levels as well as the enactment and implementation of anti FGM law and policies. Evidence suggest that Mali, a country where FGM is not legally prohibited has become a safe haven for FGM perpetrators in the neighbouring countries where FGM is prohibited such as Burkina Faso, Guinea and Senegal [36]. To make further progress in eradicating the FGM practice, laws against FGM should be enacted and enforced in all countries.

## Conclusions

The practice of FGM is declining in Burkina Faso but levels still remain high. The proportion of girls undergoing FGM before the age of five years is increasing. Factors associated with a lower likelihood of FGM include being of a younger birth cohort, being educated, urban residence, being Christian and being of some ethnic groups (Fulfuldé, Peul, Touareg, Bella, Gourmatché, Gourounsi and Bissa). In order to tackle the practice of FGM, policymakers and practitioners in Burkina Faso need to emphasise girls’ participation in education and implement programmes to sensitize and raise public awareness of the dangers of FGM. The programmes should also be targeted at high-risk groups including Muslim women, women from rural areas and women of Mossi ethnicity.

## Endnote

^a^The analysis for community level variance uses the V001 variable which represents a cluster, the final stage of selecting households to be interviewed. It is expected that women from the same clusters may share similar beliefs and/or traditional practices such female circumcision.

## Additional file

Additional file 1:
**Ethnic Map of Burkina Faso.**
