# Supplementary material for: Trends and protective factors of female genital mutilation in Burkina Faso: 1999 to 2010
Source: Int J Equity Health. 2015 May 8;14:42. doi: 10.1186/s12939-015-0171-1 (PMC4437659; doi:10.1186/s12939-015-0171-1)
Supplement: Additional file 1: — Ethnic Map of Burkina Faso. [file 12939_2015_171_MOESM1_ESM.pdf]

Additional file 1. Ethnic Map of Burkina Faso

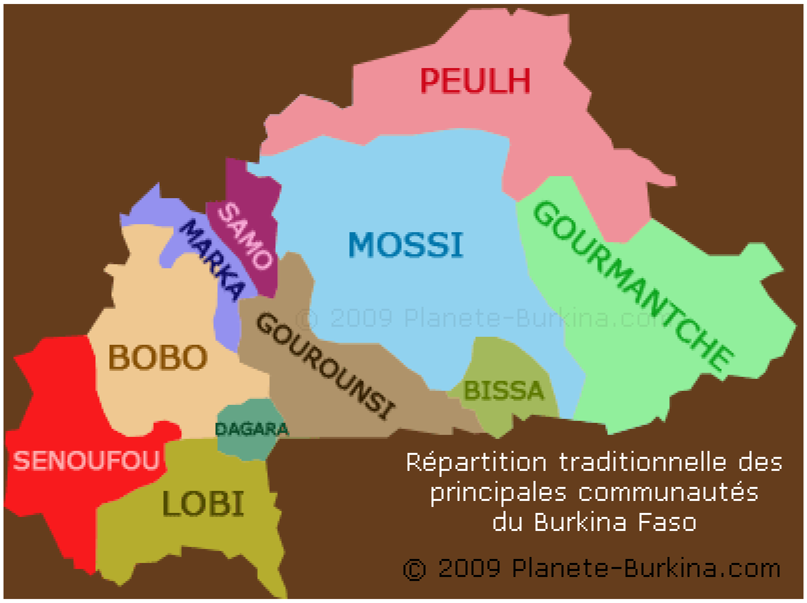

Source: http://www.planete-burkina.com/ethnies_burkina_faso.php
